# Açaí (*Euterpe oleracea*) pulp-enriched diet induces anxiolytic-like effects and improves memory retention

**DOI:** 10.29219/fnr.v66.8851

**Published:** 2022-11-02

**Authors:** Tayana Silva de Carvalho, Alódia Brasil, Luana K. R. Leão, Danielle Valente Braga, Mateus Santos-Silva, Nadyme Assad, Waldo Lucas Luz, Evander de Jesus O. Batista, Gilmara de Nazareth Tavares Bastos, Karen Renata Matos Herculano Oliveira, Domingos Luiz Wanderley Picanço Diniz, Anderson Manoel Herculano

**Affiliations:** 1 Laboratório de Neurofarmacologia Experimental, Universidade Federal do Pará, Instituto de Ciências Biológicas, Belém, Brazil; 2 Laboratório de Neurologia Tropical, Núcleo de Medicina Tropical, Universidade Federal do Pará, Belém, Brazil; 3 Núcleo de Medicina Tropical, Universidade Federal do Pará, Belém, Brazil; 4 Laboratório de Neuroinflamação, Universidade Federal do Pará, Belém, Brazil; 5 Universidade Federal do Oeste do Pará, Santarém, Brazil

**Keywords:** anxiety, memory, oxidative stress, hippocampus, polyunsaturated diet

## Abstract

**Background:**

Açaí (Euterpe oleracea) has a rich nutritional composition, showing nutraceutical and protective effects in several organs. In this study, the effects of an açaí-enriched diet on motor performance, anxiety-like behavior, and memory retention were deeply investigated.

**Methods:**

Eight-week male Wistar rats were fed with an Euterpe oleracea (EO) pulp-enriched diet, an olive oil-enriched (OO) diet (polyunsaturated fatty acid [PUFA] fat control diet), or a chow diet for 31 days (28 days pre-treatment and 3 days during behavioral tests). Afterward, animals were submitted to a battery of behavioral tests to evaluate spontaneous motor behavior (open-field test), anxiety-like behavior (elevated plus maze and open-field test), and memory retention (step-down). Oxidative stress in the hippocampus was evaluated by a lipid peroxidation assay.

**Results:**

EO-enriched diet did not influence the body weight and food intake but increased the glucose plasmatic level after 31 days under this diet. However, a similar fat-enriched diet stimulated a marked weight gain and reduced the food intake, followed by changes in the plasmatic lipid markers. EO-enriched diet preserved the motor spontaneous performance, increased the exploration in the aversive environment (anxiolytic-like effects), and elevated the latency to step-down (improved memory retention). The EO-enriched diet also reduced the level of lipid peroxidation in the hippocampus. These positive effects of EO-enriched diet can greatly support the usage of this diet as a preventive therapy.

**Conclusion:**

Taken together, the current study suggests that Euterpe oleracea-enriched diet promotes anxiolytic-like effects and improves memory consolidation, possibly due to the reduced levels of lipid peroxidation in the hippocampus.

## Popular scientific summary

- A balance diet style became one of the promising targets for preventing memory loss and/or behavioral alterations.
- Considering that these diseases are becoming more common, it is a challenge to prevent them only via pharmacological intervention.
- Therefore, we proved that Açaí is able to play this role, due to its rich nutritional and antioxidant compositions. It can help brain cells to become stronger and resistant against cell degeneration, a common feature of neurobehavioral changes.

In the last two decades, açaí fruit (*Euterpe oleracea Mart. Palmae*, Arecaceae, EO) has been highlighted as an important nutraceutical for preventing several diseases and aging processes (1–10). Particularly in the north of Brazil, this fruit is consumed as pulp, and it is part of the daily dietary habits of people from this region (2). EO pulp has an outstanding nutritional composition based on 55% calories of monounsaturated fatty acids – MUFAs and polyunsaturated fatty acids – PUFAs (11). The remaining nutrients belong to carbohydrates, dietary fibers, vitamins, anthocyanins, and flavonoids (11).

Clinical studies demonstrated that EO pulp improves vascular system response by stimulating the activity of plasmatic apolipoprotein A1 and paraoxonase-1 (high-density lipoprotein [HDL] precursors) in healthy women (12). Similarly, protective effects were observed in experimental studies, demonstrating that EO pulp not only improves the systemic vascular response but also induces anti-inflammatory and antioxidant effects even at the brain level (10, 13–15). Moreover, these positive effects on brain function were able to secondary inhibit the impairment of behavioral alterations in models of chronic tinnitus, periodic maternal separation, depression, and methylmercury exposure models (3, 6, 9, 16). Therefore, these findings indicate that EO pulp can be potentially used for improving neurobehavioral function, especially before the onset of neurodegenerative diseases (17).

The need for understanding this kind of degenerative process occurs due to, nowadays, one-third of the world population suffers from some level of neurobehavioral diseases such as anxiety disorders and memory loss (18, 19). Besides that, these two particular neurodiseases are highly interconnected or even dependent on each other in severe cases of anxiety, panic attack, and dementia (20, 21).

Therefore, to identify whether EO pulp-diet could be able to regulate the spontaneous behavior and memory retention process under a certain level of stress, healthy animals were fed with EO pulp-diet for 28 days before the behavioral tests. Additionally, we used as a PUFA control an olive oil (OO)-based diet to better investigate whether a rich polyunsaturated fat diet composition could be the main responsible for the effects induced by EO-pulp on the behavioral response. The justification for this choice is due to EO and OO have similar ratio of Omega 6:Omega 3 (10:1) in their composition.

## Material and methods

### Animals, ethical issues, and randomization

Thirty male *Wistar* rats (150–200 g), 8 weeks of age, were used for assessing the behavioral and biochemical measurements. Throughout the study, the animals were housed in groups of five animals *per* cage and maintained at a central animal facility of the Federal University of Pará (UFPa) in a 12 h:12 h light/dark cycle. Experiments were approved by the local ethics committee (*CEPAE/UFPA* 122-13) and also followed ARRIVE guidelines for *in vivo* animal experiments. All animals were placed in an inverted daily cycle, and the experiments were done in DIM lights. The experimenter was blinded by a third person, not involved in the analysis, who randomized the animals into three groups as described below. This person also provided fresh food pellets every 3 days and measured the daily food intake and weekly weight changes. Additionally, for further characterization of EO pulp effects on behavioral response, we decided to stick to male animals for avoiding hormone fluctuations that commonly affect female and old rats.

### Diet

Fresh EO (*Euterpe oleracea*) pulp was prepared using fruits obtained from the local farms. The pulp was done under the Brazilian regulation (Collegiate Directive Board Resolution or Resolução da Diretoria Colegiada [RDC] nº216 from 15/08/2004 and RDC nº12 from 02/01/01) for food preparation and manipulation. OO-based diet was assigned for being a PUFA fat control for the EO pulp diet. The oil was obtained from commercial food stores. Then, EO pulp and OO were mixed into the animal chow, originating the enriched diets. Thereafter, animals assigned to the control group received only the normal chow. In Table 1, a detailed nutritional composition from the diet mixtures (EO-enriched diet and OO-enriched diet) and normal chow was provided.

**Table 1:** Diet composition of the enriched diets and normal chow

Nutrients	100% normal chow (100 g)	10% EO pulp with 90% normal chow (100 g)	10% OO with 90% normal chow (100 g)
Total calorie (kcal/100 g)	1430.0	1336.8	1377.1
Protein (g/100 g)	220.0	198.9	198
Total fat (g/100 g)	40.0	40.9	46
PUFAs	23.2*	22.7 (Omega 6/Omega 3. 10:1)	24.5 (Omega 6/Omega 3.10:1)
Carbohydrates (g/100 g)	50.0**	45.3	45

* Fat composition based on soja oil, in which 53% of the total fat corresponds to PUFAs.

** Crude fiber represents carbohydrates.

Normal chow composition was obtained from the manufacturer. Açaí pulp composition was obtained from (1). Extra virgin Oil composition was obtained from (2). The calculation was based on 90% of the normal chow calorie with 10% of the EO or OO.

EO, Euterpe oleracea; OO, olive oil; PUFA, polyunsaturated fatty acids.

**Fig. 1:**
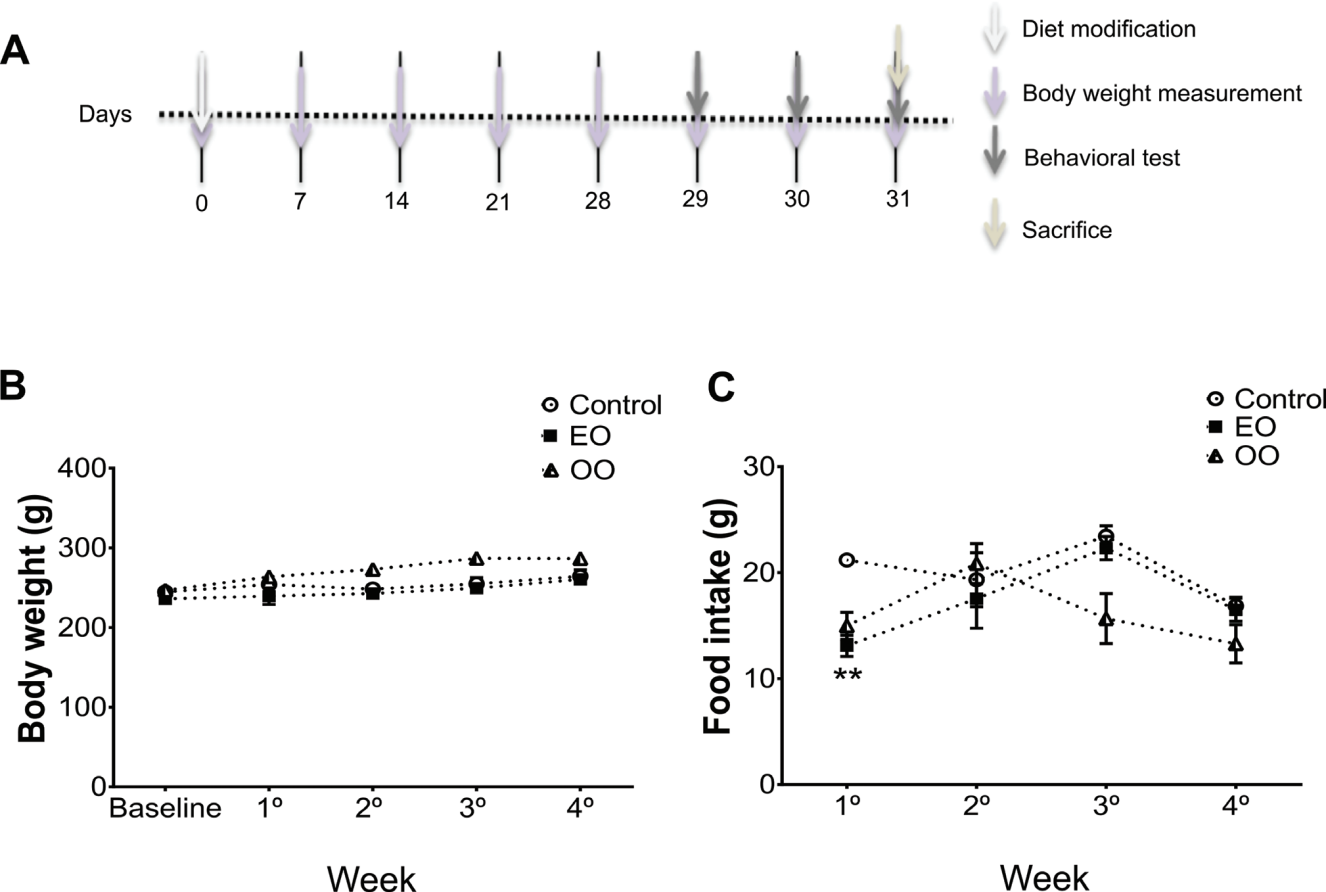
Euterpe oleracea (EO)-enriched diet did not change body weight and food intake. (A) Schematic figure illustrating the experimental design of our experimental study, (B) weekly body weight measurements (g), and (C) weekly food intake measurements (g). Data are means ± SEM values. Statistical analyses were performed by repeated measurements ANOVA followed by the Bonferroni test as a post hoc test (GraphPrism 7.0). **P < 0.01 EO-enriched diet group compared with control diet group (*n* = 10 animals/group).

The enriched foods were obtained by mixing the bran from the regular commercial food for rodents (Presence, 3.093/PURINA Company *Cia*, São Paulo, Brazil) with EO pulp (10:1 g/g diluted in ultrapure water) or OO (10:1 g/g diluted in ultrapure water). The calculation of the total calories from each enriched diet followed the dilution ratio (90% of the calorie from normal chow and 10% of the calorie from EO or OO) described above. Then, the mixture of both diets was shaped as a pellet format, baked at 40ºC for 2 h, and cooled to room temperature before being offered to animals. The food production was weekly done, and fresh pellets were replaced every 3 days. The mean consumption reported in Fig. 1C was assessed after dividing the total daily consumption per the number of animals in each cage. After that, an average between the values obtained every 7 days was done. Animals were randomized into three different groups: G1 was fed with a regular commercial rat diet (*n* = 10), G2 was fed with an EO-enriched diet (*n* = 10), and G3 was fed with an OO-enriched diet (*n* = 10) for 28 days before the behavioral tests and also during the behavioral test days, resulting in 31 days of diet intervention (Fig. 1A). Intervention time and diet concentration were chosen based on a previous study performed by our group (3).

### Open-field test

After 28 days, all the animals were submitted to a battery of behavioral tests that are described in the present study in the same sequence in which they were applied. Besides that, it is worthy to mention that no animals were excluded. Open-field test (OFT) is a circular wooden arena (52 × 52 × 30 cm) that aims to evaluate the spontaneous motor behavior (assessed by the number of squares crossed) and the level of anxiety-like behavior (assessed by the total time spent in the center or in the periphery of the apparatus). For the analysis, squares were virtually traced in the software (in which the middle ones represented the central region of the OFT), making it possible to quantify the number of squares crossed (crossing all four paws over the demarked line). Additionally, with virtual squares were possible to better determine spatial limits between the aversive or non-aversive sides. Then, the sum of the time in each of these squares at the end of the recording was performed, indicating the nature of given animal behavior. It was also possible to make a virtual representation of each animal behavior by using a colorful line (orange) that traces the total exploration in OFT. Consequently, strong tangle of lines represents higher levels of exploration in a given place. For starting this behavior test, each animal was placed (only once for evaluation of spontaneous behavior) in the center of the apparatus, and all the parameters were recorded for 3 min (3). The recording was done by a digital camera (30 fps), captured by the Debut Video Capture Software version 1.49 and analyzed by the X-Plo-Rat 2005 software (http://scotty.ffclrp.usp.br) (4).

### Elevated plus-maze

**Fig. 2:**
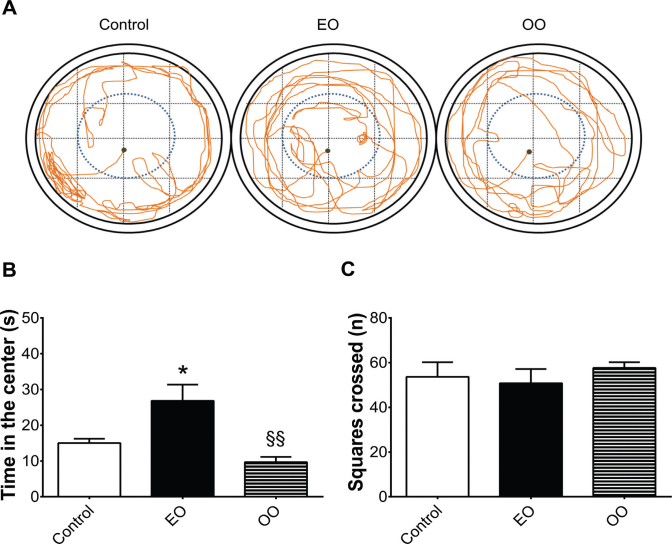
Euterpe oleracea (EO)-enriched diet did not alter the spontaneous motor behavior and increased the time in the aversive compartment of the open field test. (A) Representative figure of rat’s behavior in the OFT, (B) anxiety-like behavior measured by the time (s) spent in the center of the OFT, and (C) spontaneous motor activity assessed by the number of squares crossed in the open-field test (OFT). Data are means ± SEM values. Statistical analyses were performed by one-way ANOVA followed by the Tukey post-test (GraphPrism 7.0). *P < 0.05/EO-enriched diet group compared with control diet group/^§§^*P* < 0.01 olive oil (OO)-enriched diet compared with EO-enriched diet group (*n* = 10 animals/group).

**Fig. 3:**
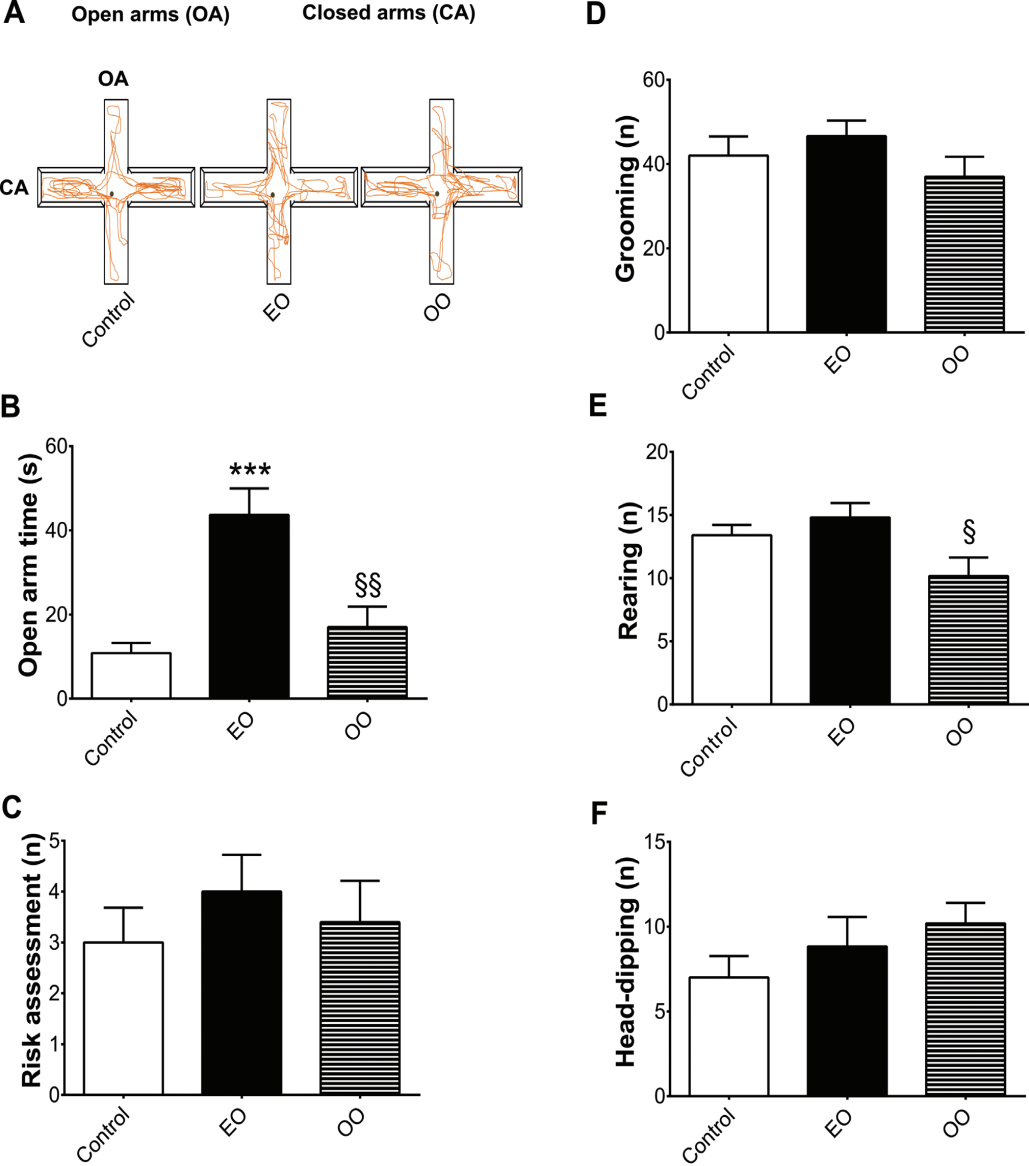
Euterpe oleracea (EO)-enriched diet-induced anxiolytic-like effects. (A) Representative figure of rat’s behavior in the elevated plus-maze (EPM), (B) anxiety-like behavior measured by the time (s) spent in the open arm, (C) number of grooming (*n*), (D) a number of rearing (*n*), (E) number of risk assessment (*n*), and (F) number of head-dipping (*n*). Data are means ± SEM values. Statistical analyses were performed by one-way ANOVA followed by the Tukey post-test (GraphPrism 7.0). ***P < 0.001/EO-enriched diet group compared with control diet group/^§^*P* < 0.05 olive oil (OO)-enriched diet compared with EO-enriched diet group (*n* = 10 animals/group).

Thirty minutes after the OFT, each animal was submitted to a plus-maze test (only once for evaluation of spontaneous behavior). This test aims to investigate anxiety-like behavior in an environment with aversive areas for rodents. Therefore, animals with longer exploration time in open area are classified with lower levels of ‘anxiogenic-like’ behavior. The elevated plus-maze (EPM) is made out of wood and has two open arms (30 × 10 cm) with 1 cm border protection, a center space, and two closed arms that are perpendicularly arranged to the open arms. Additionally, this apparatus is elevated 50 cm from the floor. At the beginning of the test, animals were individually placed in the center with the head oriented to one of the closed arms. During the experiment, their behavior was freely recorded for 3 min. For the analysis, the squares were virtually traced in the software, making it possible to determine in each side (aversive or non-aversive) of the apparatus the animals mostly explored. Additionally, a virtual representation of each animal behavior was done by using a colorful line (orange). Consequently, strong tangle of lines represents higher levels of exploration in a given place (as shown in the results section, Figs. 2A and 3A). Entries in the closed or open arms were recorded only when the animal had all four paws within one arm. ‘Ethological’ behaviors such as the frequencies of head-dipping, risk assessment, rearing, and grooming were measured. The frequency of defecation was counted, as a common sign of fear reaction from rodents against scared situations (5). The EPM test was also recorded by a digital camera (30 fps), captured by the Debut Video Capture Software version 1.49 and analyzed using the software X-Plo-Rat 2005 (http://scotty.ffclrp.usp.br) (6).

### Step-down avoidance task

One hour after the EPM test, each animal was submitted to the step-down avoidance task for measuring memory retention (24h interval protocol) (7, 8). This apparatus has an acrylic box (50×25×25), whose floor has a steel bar spaced 1.0 cm apart. Additionally, a wooden platform (7×2.5×15) is normally placed at the center of the apparatus during the training and test. For two consecutive days (training days), animals were individually placed on the platform, and the time to step down on the steel bar with four paws was quantified. During this time, directly after stepping down, animals received a 1mA/1s foot shock. Then, on the third day (testing day), the latency to step down was measured. An optimal learning curve was associated with longer latency to step down on the test day compared with the second training day (8).

### Plasma measurements and animal sacrifice

After 31 days of the experiment, animals were deeply anesthetized with ketamine (100 mg/kg)/xylazine (10 mg/kg) by intraperitoneal injection. Blood was collected in EDTA tubes by cardiac puncture. Plasma was obtained after centrifugation at 3,000 rpm for 5 min. These samples were used for the analysis of cholesterol, HDLs, low-density lipoproteins (LDL), very-low-density lipoproteins (VLDL), triglycerides, and glucose (CMD 800iX1, Wiener lab group, Rosário, Argentin). Subsequentially, animals were quickly euthanized by decapitation. The brain was harvested, and the hippocampus was dissected for being used in lipid peroxidation (LP) assay.

### LP assay

Hippocampus (a key area responsible for emotion networking and memory formation in the brain) was harvested. This tissue was manually homogenized in phosphate buffer saline pH 7.4 at 4°C (9). Homogenates were centrifuged at 3,000 rpm for 5 min. The supernatant was used for the biochemical analysis of LP through thiobarbituric acid reactive substances (TBARS) methods. As malondialdehyde (MDA) is a product of LP, a standard curve concentration of MDA degradation was used for determining LP levels (3, 6, 10). The measurement was done using absorbance at a 535 nm wavelength. MDA concentration was quantified in nmol per milligram of protein. Proteins were quantified by the Bradford method (11). All the values were expressed as a total value in μmol/g tissue.

### Statistical analysis

Statistical analyses were performed using GraphPad Prism 7.0. Bodyweight and food intake were analyzed by repeated measurements ANOVA followed by the Bonferroni test as a post hoc test. Plasma measurements and behavioral test data were analyzed by one-way ANOVA followed by the Tukey test. The LP assay was analyzed by Student’s *t*-test. All the results are presented as mean ± Standard Error of the Mean (SEM) values. *P* values < 0.05 were defined to indicate statistical significance.

## Results

### EO-enriched diet did not alter body weight and food intake, but affected glucose plasmatic levels

The nutritional assessments showed that an EO-enriched diet did not alter the body weight up to the end of the experiment (Fig. 1B). This group also had a similar food intake when compared with the normal diet group, except in the beginning of the diet intervention (Fig. 1C). Additionally, a table (Table 2) with the accumulative means of body weight and food intake was provided, for better represent the statistical differences, not included in the graphs, induced by OO-enriched diet in relation to normal diet and EO-enriched diet.

**Table 2:** Statistical results from the accumulative values of body weight and food intake obtained after 4 weeks of diet

Body weight	Normal diet	EO-enriched diet	OO-enriched diet
Baseline	244.6 ± 4.6	236.1 ± 4.8	246.6 ± 1.6 ^++ §§§^
1º Week	254.0 ± 6.3	239.3 ± 10.4	263.7 ± 2.7^++ §§§^
2º Week	248.3 ± 6.9	242.6 ± 5.6	273.0 ± 1.7^++ §§§^
3º Week	254.8 ± 8.0	249.3 ± 5.0	286.8 ± 2.4^++ §§§^
4º Week	264.3 ± 8.3	260.5 ± 5.9	286.6 ± 2.6^++ §§§^
Food intake			
1º Week	21.2 ± 0.5	13.9 ± 0.9	15.0 ± 1.3
2º Week	19.3 ± 2.5	17.5 ± 2.8	20.8 ± 1.8
3º Week	23.4 ± 0.9	22.3 ± 1.1	15.6 ± 2.3
4º Week	16.8 ± 0.8	16.5 ± 1.1	13.3 ± 1.8

Data are means ± SEM values, evaluated at 31 dpi. Statistical analyses were performed by repeated measurements ANOVA followed by the Tukey post-test (GraphPrism 7.0). ***P < 0.001 EO-enriched diet compared with normal diet;

§§§ *P* < 0.01 OO-enriched diet compared with EO-enriched diet;

++ *P* < 0.01 OO-enriched diet compared with control diet (*n* = 10 animals/group).

EO, Euterpe oleracea; OO, olive oil.

The nutritional biomarkers revealed that an EO-enriched diet increased serum glucose levels when compared with the normal diet group (124 ± 1.75 *vs* 94.6 ± 1.8 mg/dl, *F*(2,15) = 3.682, *P* < 0.001) and when compared with OO-enriched diet group (124 ± 1.75 *vs* 91.5 ± 3.3 mg/dl, *F*(2,15) = 3.682, *P* < 0.001) (Table 3). Surprisingly, the plasmatic levels of lipid metabolism markers did not follow the glucose parameters in animals fed with EO-enriched. Contrariwise, the PUFA fat control diet (OO-enriched diet) effectively affects the cholesterol levels (81 ± 6.7 *vs* 59.8 ± 2.7 mg/dl, *F*(2,15) = 11.04/*P* < 0.05) when compared with EO-enriched diet and LDL (24.1 ± 1.9 *vs* 21.2 ± 3.9 mg/dl, *F*(2,15) = 6.623/*P* < 0.01), HDL (33.8 ± 1.2 *vs* 27.3 ± 1.2, *F*(2,15) = 5.807/*P* < 0.05), cholesterol levels (81 ± 6.7 *vs* 52.2 ± 2.7 mg/dl, *F*(2,15) = 11.04/*P* < 0.01), and triglycerides levels (34.3 ± 3.5 *vs* 53.3 ± 7.4 mg/dl, *F*(2,15) = 3.485/*P* < 0.05) when compared with normal diet (Table 3).

**Table 3:** Blood parameters changes induced by enriched diets

Parameters	Normal diet	EO-enriched diet	OO-enriched diet
Cholesterol (mg/dl)	52.1 ± 2.7	59.8 ± 2.7	81.0 ± 6.7^§ ++^
LDL (mg/dl)	14.1 ± 2.0	21.1 ± 1.6	24.1.0 ± 1.9^++^
HDL (mg/dl)	27.3 ± 1.2	28.0 ± 1.8	33.8 ± 0.8^+§^
VLDL (mg/dl)	10.6 ± 1.5	9.0 ± 0.8	6.8 ± 0.6
Triglycerides (mg/dl)	55.2 ± 7.4	44.0 ± 3.2	34.3 ± 3.5^+^
Glucose (mg/dl)	94.6 ± 1.8	124.0 ± 1.75***	91.5 ± 3.3^§§§^

Data are means ± SEM values, evaluated at 31 dpi. Statistical analyses were performed by one-way ANOVA, followed by the Tukey post-test (GraphPrism 7.0).

*** *P* < 0.001 EO-enriched diet compared with normal diet;

§§§ *P* < 0.01/

§ *P* < 0.05 OO-enriched diet compared with EO-enriched diet;

++ *P* < 0.01/

+ *P* < 0.05 OO-enriched diet compared with control diet (*n* = 10 animals/group).

LDL, low-density lipoprotein; HDL, high-density lipoprotein; VLDL, very low-density lipoprotein; EO, Euterpe oleracea; OO, olive oil.

### EO-enriched diet-induced anxiolytic-like effects and improved memory retention

The battery of behavioral tests revealed that animals fed with an EO-enriched diet increased the time in the aversive compartments (open zone) of the OFT (*F*(2,16) = 3.634/*P* < 0.05) (Fig. 2A and B) and of the EPM apparatus (open arms) (*F*(2,14) = 3.739/*P* < 0.001), when compared with animals fed with normal diet, meaning anxiolytic-like effects (Fig. 3A and B). Representative figures confirmed these results, showing strong tangle of orange lines in the aversive compartments (anxiolytic-like effects) induced by EO-enriched group when compared with normal diet group (Figs. 2A and 3A).

Furthermore, anxiogenic-like parameters such as risk assessment (*P* > 0.05) (Fig. 3C), grooming (*P* > 0.05) (Fig. 3D), rearing (*P* > 0.05) (Fig. 3E), and head-dipping (*P* > 0.05) (Fig. 3F) were not affected by EO-enriched group when compared with the control group. Additionally, regarding diet intervention, none of the animals showed changes in the frequency of defecation (data not shown).

On the other hand, OO-enriched diet did not differ from the normal diet group but showed slightly reduction of exploration time in the aversive part of the open field (*F*(2,16) = 5.608/*P* < 0.01) (Fig. 2B) and in the EPM (*F*(2,14) = 5.435/*P* < 0.01) when compared with EO-enriched diet (Fig. 3B). Additionally, it was found a reduction in the number of rearing behavior ((*F*(2,14) = 4.633/(*P* < 0.05)) when compared with an EO-enriched diet (Fig. 3E). Based on these data, we assume that OO-enriched diet was unable to reproduce the anxiolytic effects induced by EO-enriched diet, even having similar PUFA ratio composition.

Thereafter, the number of crossed squares in the open field (Fig. 2C) and the frequency of entrance in the open and closed arms of the EPM were not altered by any of the enriched diets (Table 4).

**Table 4:** Behavioral parameters induced by enriched diets

Parameters	Normal diet	EO-enriched diet	OO-enriched diet
Frequency of open arms entrance (*n*)	3.0 ± 0.5	2.3 ± 0.4	4.6 ± 0.7^§^
Frequency of closed arms entrance (*n*)	3.3 ± 0.5	2.6 ± 0.4	3.2 ± 0.4

Data are means ± SEM values, evaluated at 31 dpi. Statistical analyses were performed by one-way ANOVA followed by the Tukey post-test (GraphPrism 7.0).

§ *P* < 0.05 (*F* = 3,701) compared with an EO-enriched diet (*n* = 10 animals/group).

EO, Euterpe oleracea; OO, olive oil.

**Fig. 4:**
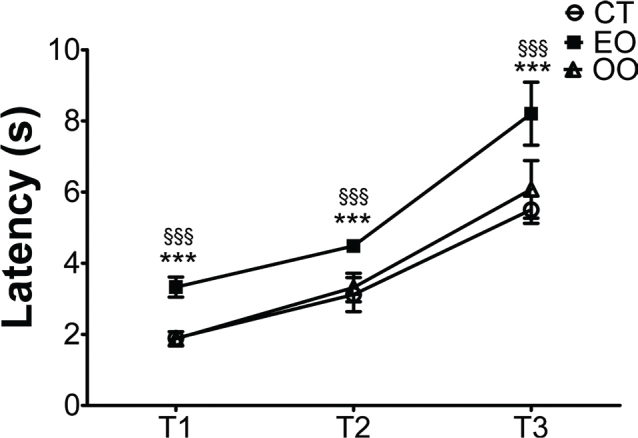
The Euterpe oleracea (EO)-enriched diet improved memory retention. (A) Memory consolidation was assessed by a step-down avoidance test. T1 – the first day of training/T2 – the second day of training/T3 – testing day. Data are means ± SEM values. Statistical analyses were performed by repeated measurements ANOVA followed by the Bonferroni test as a post hoc test (GraphPrism 7.0). ***P < 0.001, EO-enriched diet group effect compared with control diet group, and ^§§§^*P* < 0.001, EO-enriched diet group effect compared with olive oil (OO)-enriched diet (*n* = 10 animals/group).

Due to the important role of anxiety on memory formation, the next step was to investigate whether the enriched diets could regulate memory retention after aversive situations. Our findings showed that all animals, regardless of the diet, were able to learn to step down from the platform. However, only the EO-enriched group showed greater memory retention (longer latency) (*F*(2,32) = 17.95/*P* < 0.001) when compared with the normal diet group and with the OO-enriched diet (*P* < 0.001) (Fig. 4A).

### EO-enriched diet prevented LP in the hippocampus

**Fig. 5:**
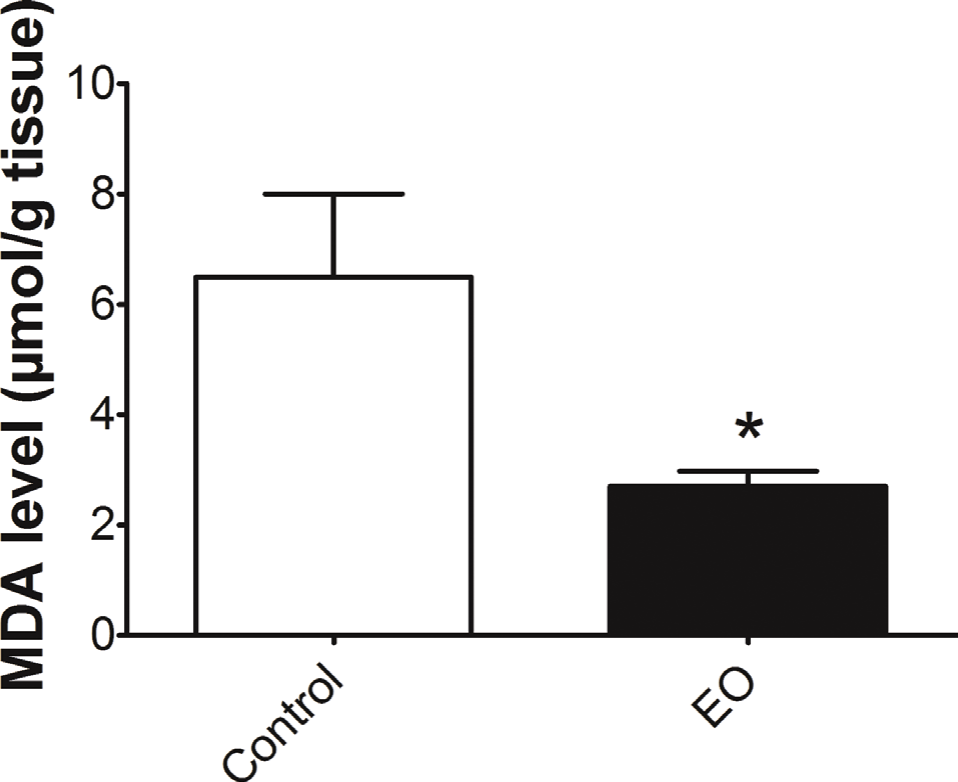
The Euterpe oleracea (EO)-enriched diet reduced lipid peroxidation. Malondialdehyde levels were determined, and the values were expressed as absolute values. Data are means ± SEM values, and statistical analyses were performed by *T*-test (GraphPrism 7.0). *P < 0.05 EO-enriched diet compared with the control group (*n* = 10 animals/group).

After submitting all the animals to different ‘new’ and aversive environments, substantial neurobehavioral improvements were observed only in animals fed with an EO-enriched diet. Then, we decided to check whether antioxidant response at the level of the hippocampus could be an explanation for neurobehavioral improvements promoted by an EO-enriched diet. Thus, we found that an EO-enriched diet was able to substantially reduce the MDA levels (*P* < 0.05) in the hippocampus of those animals when compared with normal diet-fed animals (Fig. 5).

## Discussion

Currently, EO pulp is not just a regional food from the North of Brazil but became a famous soft drink worldwide (12). Apart from the incomparable flavor, neuroprotective effects were already reported in different disease models, reinforcing the need to explore the potential effects of this fruit on health. In this context, our group is making major contributions to understanding the role of the EO-pulp diet on methylmercury intoxication and cerebral malaria, which are important health public problems in Brazil (3, 13). We demonstrated that an EO-enriched diet prevented retinal oxidative and functional damage associated with methylmercury intoxication (3). As well, this diet was able to reduce mortality, protect the blood–brain barrier, and reduce the neurocognitive effects of animals infected with *Plasmodium berghei* (ANKA) (13). Based on these studies, we decided to drive our focus to understanding the role of this diet in neurobehavioral changes, since they are emerging as a worldwide public health problem.

Hence, in the present study, we demonstrated, for the first time, that an EO-enriched diet can induce anxiolytic-like effects and improve memory retention in healthy animals submitted to aversive conditions. Unfortunately, these positive alterations could not be explained by the improvement of metabolic parameters. Since long-term EO-enriched was unable to improve the lipid markers and nutritional parameters as well as to regulate glucose levels in healthy animals. On the other hand, an OO-enriched diet significantly impaired these lipid metabolic markers (except the triglycerides levels), showing that a diet solely based on PUFAs composition can also fail to maintain greater metabolic response in the long term. Nevertheless, in terms of mechanism, the EO-enriched diet might be supported by the greater antioxidant activity that was observed at the level of the hippocampus.

Previously, it was reported that EO pulp can lower cholesterol, LDL levels, and post-prandial glucose in obese humans (14). Interestingly, our experimental study did not show similar reductions, most probably because EO-enriched is unable to play such a role in eutrophic animals. On the other hand, after 31 days of the diet, the chosen control (based on OO) diet seems to drive the metabolism in the direction of a metabolic syndrome. This means that even have a proper amount of PUFA, this diet failed to improve nutritional parameters, due to the other kind of fats (saturated fatty acids and omega 9) in its composition (15). A similar nutritional and metabolic observation was done by Keita et al., who observed that 20 weeks of OO diet induces weight gain and insulin resistance (16). Nevertheless, by comparing the 4-week intervention in our study and 20-week experiment from Keita and colleagues, we assume that OO does not need such long time to negatively change the lipid metabolism response.

Previous studies indicated that higher levels of plasmatic glucose alter insulin and IGF1 receptors activities in different brain regions, such as the hippocampus, boosting the onset of anxiety and memory loss (17, 18). However, the antioxidant composition and the further nutritional compounds of EO-pulp can modulate the levels of these glucose metabolic markers, lowering the chances of the onset of neurobehavioral disease (18, 19).

To our knowledge, several studies have already shown the beneficial effect of fruits on the prevention of anxiety disorder and memory loss. They observed that healthy animals fed with buriti (*Mauritia flexuosa*), fig (*genus Ficus*), and banana (*Musa sapientum L*.) fruit pulps, and peel extract spent more time in aversive environments and showed improved spatial memory, learning, and psychomotor coordination (6, 20, 21). Curiously, even having a different nutritional composition, the level of oxidants among these fruits was enough to assure the potential neurobehavioral effects, being a possible support therapy for neurological disorders.

Particularly about EO-pulp, only a few studies investigated the neurobehavioral effects associated with this fruit. They found that EO-pulp was able to suppress depressive-like behavior, decrease the expression of TERT in the hippocampus, striatum, and prefrontal cortex, and prevent neuronal loss in LPS-induced depressive-like behavior model (22). However, in terms of cognition, we observed contradictory results in the literature. On the one hand, EO extracts can inhibit the β-amyloid aggregation in cells, being a potential therapeutic agent for treating Alzheimer’s disease (23). On the other hand, cotreatment with high doses (100 mg/kg and 300 mg/kg) of EO and antidepressants did not influence learning and memory consolidation in healthy animals (24).

Interestingly, our findings showed that animals fed with an EO-enriched diet freely explored the aversive environments in two different paradigms (open and elevated areas). Thereafter, the animals positively increased the latency to step down even after being under such aversive conditions. This latter finding strongly indicated that EO pulp might behave as a ‘comfort food’, increasing the feeling of well-being even after an aversive experience. On the other hand, the OO diet was unable to induce similar effects.

Mechanistically, the neurobehavioral response induced by the EO-enriched group could be better associated with its antioxidant properties than its rich composition of PUFA, since OO enriched diet (comparable PUFA diet) failed to have similar results. Since that our findings revealed low MDA levels in the hippocampus without being followed by lipid metabolism improvements, previous studies proved that polyphenols from EO reduce LP and ROS production, increase the activity of endogenous antioxidant enzymes such as glutathione peroxidase (GPX) and superoxide dismutase (SOD), and lower the expression of pro-inflammatory markers (3, 22, 25, 26). This fruit can also boost cells’ energy and the microbiome-gut-brain axis activity, modulate neurotransmitter levels, recruit anti-inflammatory markers, and induce antioxidant response (20, 24, 25). At the hippocampus level, EO-pulp modulates nuclear factor-erythroid factor 2-related factor 2 (Nrf-2) and ubiquitin-proteasomal pathway, slowing down aging processes (27). However, Poulose and colleagues did not conduct a behavioral test to deeply correlate molecular changes with potential neurobehavioral improvements (27). Based on these studies, we can assume that this is the first study to associate the anxiolytic-like behaviors and greater memory retention with lower MDA levels in the hippocampus of animals fed with an EO-enriched diet.

The strengths of this study were the well-controlled nutritional assessments and the use of a battery of behavioral tests and biochemical assays. This study indicates that an EO-enriched diet has profound consequences on neurobehavioral changes and can be a possible non-pharmacological intervention for preventing anxiety and memory loss.

However, some limitations of this study must be addressed such as: 1) OO diet is a limited fat control, due to induce metabolic alterations; 2) our descriptive results offer the basis for understanding behavioral changes and partially biochemistry alterations, but it still lacks more functional and molecular data, being, therefore, the next focus of our group; 3) future studies with the same experimental setting using females, obese, and aging animals need to be conducted, to broadly understand the effects of this diet on neurobehavioral changes in all these groups.

## Conclusion

This study showed that an EO-enriched diet can protect the brain by offering proper nutrients and antioxidants sources. Besides that, EO-enriched showed a potential non-pharmacological effect, being an approachable intervention for controlling anxiety levels and for boosting memory retention. This is an important breakthrough for neurobehavioral treatments, once the current therapies are not fully effective and might need functional diets to support them. However, further studies are warranted for a better understanding of the molecular mechanisms associated with EO effects in neurobehavioral changes.

## Data Availability

The original contributions shown in this study are included in the present paper; further inquiries can be addressed to the corresponding author.

## References

[1] Oliveira AR, Ribeiro AEC, Oliveira ÉR, Garcia MC, Soares Júnior MS, Caliari M. Structural and physicochemical properties of freeze-dried açaí pulp (Euterpe oleracea Mart.). Food Sci Technol 2020; 40(2): 282–9. doi: 10.1590/fst.34818

[2] Mazzocchi A, Leone L, Agostoni C, Pali-Scholl I. The secrets of the Mediterranean diet. Does [only] olive oil matter? Nutrients 2019; 11(12): 1–14. doi: 10.3390/nu11122941 PMC6949890 31817038

[3] Brasil A, Rocha FAF, Gomes BD, Oliveira KRM, de Carvalho TS, Batista EJO, et al. Diet enriched with the Amazon fruit acai (Euterpe oleracea) prevents electrophysiological deficits and oxidative stress induced by methyl-mercury in the rat retina. Nutr Neurosci 2017; 20(5): 265–72. doi: 10.1080/1028415X.2015.1119378 26863909

[4] Seibenhener ML, Wooten MC. Use of the open field maze to measure locomotor and anxiety-like behavior in mice. J Vis Exp 2015; (96): e52434. doi: 10.3791/52434 25742564 PMC4354627

[5] Natalia S, Angelika P, Michal K, Iveta B. Determination of motor activity and anxiety-related behaviour in rodents: methodological aspects and role of nitric oxide. Interdiscip Toxicol 2013; 6(3): 126–35. doi: 10.2478/intox-2013-0020 24678249 PMC3967438

[6] Leao LKR, Herculano AM, Maximino C, Brasil Costa A, Gouveia A, Jr., Batista EO, et al. Mauritia flexuosa L. protects against deficits in memory acquisition and oxidative stress in rat hippocampus induced by methylmercury exposure. Nutr Neurosci 2017; 20(5): 297–304. doi: 10.1080/1028415X.2015.1133030 26869022

[7] Walz R, Roesler R, Barros DM, de Souza MM, Rodrigues C, Sant’Anna MK, et al. Effects of post-training infusions of a mitogen-activated protein kinase kinase inhibitor into the hippocampus or entorhinal cortex on short- and long-term retention of inhibitory avoidance. Behav Pharmacol 1999; 10: 723–30. doi: 10.1097/00008877-199912000-00003 10780287

[8] Borba Filho GL, Zenki KC, Kalinine E, Baggio S, Pettenuzzo L, Zimmer ER, et al. A new device for step-down inhibitory avoidance task – effects of low and high frequency in a novel device for passive inhibitory avoidance task that avoids bioimpedance variations. PLoS One 2015; 10(2): 1–17. doi: 10.1371/journal.pone.0116000 PMC4338061 25706879

[9] Zhu Y, Gao H, Tong L, Li Z, Wang L, Zhang C, et al. Emotion regulation of hippocampus using real-time fMRI neurofeedback in healthy human. Front Hum Neurosci 2019; 13: 242. doi: 10.3389/fnhum.2019.00242 31379539 PMC6660260

[10] Uchiyama M, Mihara M. Determination of malonaldehyde precursor in tusses by thiobarbituric acid test. Anal Biochem 1978; 86: 217–78. doi: 10.1016/0003-2697(78)90342-1 655387

[11] Bradford MM. A rapid and sensitive method for the quantitation of microgram quantities of protein utilizing the principle of protein-dye binding. Anal Biochem 1976; 72: 248–54. doi: 10.1016/0003-2697(76)90527-3 942051

[12] Heinrich M, Dhanji T, Casselman I. Açai (Euterpe oleracea Mart.) – a phytochemical and pharmacological assessment of the species’ health claims. Phytochem Lett 2011; 4(1): 10–21. doi: 10.1016/j.phytol.2010.11.005

[13] Oliveira K, Torres MLM, Kauffmann N, de Azevedo Ataide BJ, de Souza Franco Mendes N, Dos Anjos LM, et al. Euterpe oleracea fruit (Acai)-enriched diet suppresses the development of experimental cerebral malaria induced by Plasmodium berghei (ANKA) infection. BMC Complement Med Ther 2022; 22(1): 11. doi: 10.1186/s12906-021-03495-9 35016657 PMC8751313

[14] Udani JK, Singh BB, Singh VJ, Barrett ML. Effects of Açai (Euterpe oleracea Mart.) berry preparation on metabolic parameters in a healthy overweight population: a pilot study. Nutr J 2011; 10: 1–7. doi: 10.1186/1475-2891-10-45 21569436 PMC3118329

[15] Jimenez-Lopez C, Carpena M, Lourenço-Lopes C, Gallardo-Gomez M, Lorenzo JM, Barba Francisco J, et al. Bioactive compounds and quality of extra virgin olive oil. Foods 2020; 9(8): 1–31. doi: 10.3390/foods9081014 PMC7466243 32731481

[16] Keita H, Juan ER-S, Paniagua-Castro N, Garduño-Siciliano L, Quevedo L. The long-term ingestion of a diet high in extra virgin olive oil produces obesity and insulin resistance but protects endothelial function in rats: a preliminary study. Diabetol Metab Syndr 2013; 5(53): 1–10. doi: 10.1186/1758-5996-5-53 24330822 10.1186/1758-5996-5-53 PMC3848810

[17] Soto M, Cai W, Konishi M, Kahn CR Insulin signaling in the hippocampus and amygdala regulates metabolism and neurobehavior. PNAS 2019; 13: 6379–84. doi: 10.1073/pnas.1817391116 PMC6442573 30765523

[18] Shukitt-Hale B, Lau FC, Carey AN, Galli RL, Spangler EL, Ingram DK, et al. Blueberry polyphenols attenuate kainic acid-induced decrements in cognition and alter inflammatory gene expression in rat hippocampus. Nutr Neurosci 2008; 11(4): 172–82. doi: 10.1179/147683008X301487 18681986 PMC5015125

[19] de Bem GF, Costa CA, Santos IB, Cristino Cordeiro VDS, de Carvalho L, de Souza MAV, et al. Antidiabetic effect of Euterpe oleracea Mart. (acai) extract and exercise training on high-fat diet and streptozotocin-induced diabetic rats: a positive interaction. PLoS One 2018; 13(6): 1–19. doi: 10.1371/journal.pone.0199207 PMC6007924 29920546

[20] Samad N, Muneer A, Ullah N, Zaman A, Ayaz MM, Ahmad I. Banana fruit pulp and peel involved in antianxiety and antidepressant effects while invigorate memory performance in male mice: possible role of potential antioxidants. Pak J Pharm Sci 2017; 30(3): 989–95. 28655697

[21] Subash S, Essa MM, Braidy N, Al-Jabri A, Vaishnav R, Al-Adawi S, et al. Consumption of fig fruits grown in Oman can improve memory, anxiety, and learning skills in a transgenic mice model of Alzheimer’s disease. Nutr Neurosci 2016; 19(10): 475–83. doi: 10.1179/1476830514Y.0000000131 24938828

[22] Souza-Monteiro JR, Arrifano GPF, Queiroz A, Mello BSF, Custodio CS, Macedo DS, et al. Antidepressant and antiaging effects of acai (Euterpe oleracea Mart.) in mice. Oxid Med Cell Longev 2019; 2019: 1–17. doi: 10.1155/2019/3614960 PMC6681600 31428223

[23] Wong DY, Musgrave IF, Harvey BS, Smid SD. Acai (Euterpe oleraceae Mart.) berry extract exerts neuroprotective effects against beta-amyloid exposure in vitro. Neurosci Lett 2013; 556: 221–6. doi: 10.1016/j.neulet.2013.10.027 24161892

[24] Bear TLK, Dalziel JE, Coad J, Roy NC, Butts CA, Gopal PK. The role of the gut microbiota in dietary interventions for depression and anxiety. Adv Nutr 2020; 11(4): 890–907. doi: 10.1093/advances/nmaa016 32149335 PMC7360462

[25] Schauss AG. The effect of acai (Euterpe spp.) fruit pulp on brain health and performance. In: Ronald Watson VP, ed. Bioactive nutraceuticals and dietary supplements in neurological and brain disease. Oxford, UK; Academic Press; 2015, pp. 179–86.

[26] Carey AN, Miller MG, Fisher DR, Bielinski DF, Gilman CK, Poulose SM, et al. Dietary supplementation with the polyphenol-rich acai pulps (Euterpe oleracea Mart. and Euterpe precatoria Mart.) improves cognition in aged rats and attenuates inflammatory signaling in BV-2 microglial cells. Nutr Neurosci 2017; 20(4): 238–45. doi: 10.1080/1028415X.2015.1115213 26618555

[27] Poulose SM, Bielinski DF, Carey A, Schauss AG, Shukitt-Hale B. Modulation of oxidative stress, inflammation, autophagy and expression of Nrf2 in hippocampus and frontal cortex of rats fed with acai-enriched diets. Nutr Neurosci 2017; 20(5): 305–15. doi: 10.1080/1028415X.2015.1125654 26750735

